# Effects of sodium tanshinone IIA sulfonate injection on pro-inflammatory cytokines, adhesion molecules and chemokines in Chinese patients with atherosclerosis and atherosclerotic cardiovascular disease: a meta-analysis of randomized controlled trials

**DOI:** 10.3389/fcvm.2025.1511747

**Published:** 2025-02-13

**Authors:** Fan Xie, Xiaoying Fu, Wenbo Li, Yujin Bao, Feng Chang, Yun Lu, Yuqiong Lu

**Affiliations:** School of International Pharmaceutical Business, China Pharmaceutical University, Nanjing, Jiangsu, China

**Keywords:** sodium tanshinone IIA sulfonate, atherosclerosis, arteriosclerotic cardiovascular disease, meta-analysis, cardiovascular

## Abstract

**Background:**

Inflammation, as the basic pathogenic mechanism of atherosclerosis, promotes the development of atherosclerosis (AS) and atherosclerotic cardiovascular disease (ASCVD). In numerous experiments based on animal and cellular models, sodium tanshinone IIA sulfonate (STS) injection has been found to reduce the levels of pro-inflammatory cytokines, adhesion molecules, and chemokines in patients with AS and ASCVD, exerting an anti-inflammatory effect to treat the disease.

**Objectives:**

This study aimed to perform a meta-analysis of randomized controlled trials (RCTs) to quantify the effects of STS on pro-inflammatory cytokines, adhesion molecules, and chemokines in patients with AS and ASCVD.

**Methods:**

Eight literature databases were searched from inception to January 2024, including PubMed, Web of Science, Cochrane Library, Ebsco, CNKI, VIP, WanFang Data, and ClinicalTrails.gov. Two reviewers independently screened articles and extracted data. The quality of the included studies was assessed using the Cochrane Risk Assessment Tool 2.0. Meta-analysis was performed using RevMan 5.4 software.

**Results:**

Of the 2,698 publications screened, 42 studies were included, and the related trials involved 4,654 Chinese patients. The meta-analysis showed that STS significantly reduced the concentration level of pro-inflammatory cytokines interleukin 6 (IL-6) [standardized mean difference (SMD)=−1.50, 95%CI(−2.06, −0.95), *p* < 0.00001], tumor necrosis factor-α (TNF-α) [SMD = −2.55, 95%CI(−3.24, −1.86), *p* < 0.00001], and interleukin-1β (IL-1β) [SMD = −1.21, 95%CI(−2.41, −0.01), *p* < 0.0001], of adhesion molecules intercellular adhesion molecule-1 (ICAM-1) [SMD = −1.28, 95%CI(−1.55, −1.02), *p* < 0.00001] and p-selectin [SMD = −1.06, 95%CI(−1.46, −0.67), *p* < 0.00001], and of chemokines fractalkine [SMD = −1.32, 95%CI(−2.02, −0.61), *p* = 0.0003] and monocyte chemoattractant protein-1 (MCP-1) [SMD = −0.83, 95%CI(−1.11, −0.55), *p* < 0.00001] among patients with AS and ASCVD.

**Conclusion:**

The use of STS in patients with AS and ASCVD appeared to significantly decrease levels of pro-inflammatory cytokines, adhesion molecules, and chemokines.

**Systematic Review Registration**: [https://www.crd.york.ac.uk/PROSPERO/], PROSPERO [CRD42024496960].

## 1 Introduction

Atherosclerosis (AS) is a progressive disease caused by repetitive damage to the arterial wall, characterized by the accumulation of lipids and fibrous elements in the arterial wall. AS can affect medium and large arteries of the brain, heart, kidneys, other vital organs, and legs, and its continued progression can lead to arteriosclerotic cardiovascular disease (ASCVD) (1, 2). ASCVD mainly includes acute coronary syndrome, stable or unstable angina pectoris, coronary artery disease, revascularization, ischemic cardiomyopathy, stroke, transient ischemic attack, peripheral arterial atherosclerotic disease, and other diseases (1, 3). ASCVD is the most important factor contributing to the growing burden of cardiovascular disease (4). Each year, approximately 20 million people die of ASCVD globally (5). Moreover, cardiovascular disease also results in a heavy burden of disease in China. According to the study of Zhang J et al. based on Global Burden of Disease (6), in 2019, the prevalence of CVD in China reached 120 million, representing a 140.02% increase since 1990. The number of deaths due to CVD in China was 4.58 million, with ASCVD contributing significantly to these deaths. The burden of ASCVD is expected to continue increasing, highlighting the urgent need for effective prevention and management strategies.

AS is considered to be an inflammatory disease. Inflammation, as its basic pathogenic mechanism, is involved in the occurrence and progression of atherosclerosis and its events (7). Recent studies have emphasized the critical role of inflammatory mechanisms in ASCVD, highlighting the importance of targeting inflammation as a therapeutic strategy. For example, the NLRP3 inflammasome, a multiprotein complex that drives the inflammatory response, has emerged as a critical contributor to ASCVD (8). Its activation triggers the production of pro-inflammatory cytokines, which promotes plaque instability and increase the risk of cardiovascular events (9). Moreover, landmark clinical trials, including CANTOS, COLCOT, and LoDoCo2, have demonstrated the efficacy of targeted inflammatory therapies in reducing cardiovascular event risk in patients with ASCVD (10–12). Notably, the CANTOS trial showed that canakinumab, an inhibitor of interleukin-1β (IL-1β), significantly reduced the incidence of recurrent cardiovascular events (10). These findings highlight the therapeutic potential of targeting specific inflammatory pathways to treat ASCVD (13). Complex mixtures such as pro-inflammatory cytokines, adhesion molecules, and chemokines play a role in maintaining and enhancing local inflammation, which promotes lesion progression (14–16). Pro-inflammatory cytokines, including interleukin-6 (IL-6), tumor necrosis factor-α (TNF-α), and interleukin-1β (IL-1β) (17), participate in almost all steps of atherosclerotic inflammation, including endothelial dysfunction in the early phase, aberrant macrophage activation in the middle phase, and formation and disruption of vulnerable plaque in the advanced phase (18). Adhesion molecules, including intercellular adhesion molecule-1 (ICAM-1) and p-selectin, promote leukocyte migration within the vascular lumen and its adhesion to vascular endothelial cells (18). Chemokines, including fractalkine and monocyte chemoattractant protein-1 (MCP-1), chemotaxis and activation of leukocytes (19). Activated leukocytes migrate from blood vessels and aggregate into affected tissues, a process co-regulated by pro-inflammatory cytokines, adhesion molecules, and chemokines (19). At the same time, factors interact with each other, leading to the progression of atherosclerosis. For example, the pro-inflammatory cytokine IL-1β induces the expression of the adhesion molecule ICAM-1, which promotes its migration between leukocytes and endothelial cells (20), and the pro-inflammatory cytokine TNF-α is an early stimulus for the production of chemokine production (19). It can be seen that the key to treating AS and ASCVD is to block the inflammatory mechanism in the development of atherosclerosis by reducing the levels of pro-inflammatory cytokines, adhesion molecules, and chemokines.

Salvia miltiorrhiza, a plant in the labiform family, is one of the most widely used and oldest traditional Chinese medicines and is commonly used in the treatment of cardiovascular diseases (21). Tanshinone IIA (Tan IIA) is a fat-soluble active component of Salvia miltiorrhiza and a monomer of traditional Chinese medicine with a variety of benefits for the cardiovascular system (22). Numerous experiments based on animal and cellular models have shown that Tan IIA can inhibit pro-inflammatory cytokines, such as IL-6 (23), TNF-α, and IL-1β (24), adhesion molecules, such as ICAM-1 and p-selectin (25), and chemokines, such as MCP-1 (26) and fractalkine (27), through multiple mechanisms, including downregulation of the NF-κB pathway, suppression of the NLRP3 inflammasome, and modulation of the MAPK/HIF-1α signaling pathway. These actions collectively contribute to its anti-inflammatory effects, which has great potential in the management and control of AS and ASCVD. Due to the poor intestinal absorption and slow action onset of Tan IIA, sodium tanshinone IIA sulfonate (STS) injection has been developed to improve the bioavailability of Tan IIA (28), and it has become the most widely used clinical formulation of Tan IIA in China (29). Compared with other traditional Chinese medicine injections formulated with Salvia miltiorrhiza extract, there were fewer adverse drug reactions and a higher safety profile (30). Unlike traditional anti-inflammatory drugs such as non-steroidal anti-inflammatory drugs (NSAIDs) and corticosteroids, which often have significant side effects, STS may offer better tolerability by reducing gastrointestinal side effects and immune suppression. According to the study by Wang et al. (31), the primary adverse reaction to STS is allergic reactions. This may be attributed to its injectable administration route and the fact that Tan IIA is an extract of Salvia miltiorrhiza, which, due to the complexity of its components and insufficient purity of effective monomers during the extraction process, can lead to the production of allergic media. Therefore, close monitoring and timely intervention are required when using STS to avoid serious consequences. Overall, STS modulates inflammation by targeting specific molecular pathways without significant severe side effects. Additionally, STS has demonstrated potential in mitigating oxidative stress and modulating immune cell activity (30), thereby expanding its therapeutic applications to chronic inflammatory diseases such as AS and ASCVD.

While STS has been widely used, clinical evidence regarding its anti-inflammatory effects has been inconsistent., no definitive conclusions can be drawn as to whether STS can significantly reduce the levels of pro-inflammatory cytokines, adhesion molecules, and chemokines in Chinese patients with AS and ASCVD. The result of a randomized controlled trial (RCT) by Li et al. (32) showed that STS did not significantly affect the level of TNF-α in patients with coronary artery disease (CAD), while another study by Xu Tao et al. (33) showed that STS could significantly reduce the level of TNF-α in patients with CAD. A meta-analysis by Kan et al. (34) showed that STS could significantly reduce the level of TNF-α in patients with acute coronary syndromes who underwent percutaneous coronary intervention (PCI), while there was insufficient evidence to show that STS reduced the level of IL-6. A systematic review by Yu et al. (35) showed that RCTs showed STS could significantly reduce the level of IL-6.

Given these conflicting findings, it is essential to conduct a comprehensive meta-analysis to systematically review and quantify the effects of STS on pro-inflammatory cytokines, adhesion molecules, and chemokines in Chinese patients with AS and ASCVD to determine the impact of STS on the inflammatory process of atherosclerosis, thereby providing a more definitive assessment of its therapeutic potential.

## 2 Materials and methods

The study's review protocol was prospectively registered at PROSPERO (No. CRD42024496960, https://www.crd.york.ac.uk/PROSPERO/) and was conducted according to the Preferred Reporting Items for Systematic Review and Meta-Analyses (PRISMA).

### 2.1 Data sources and search strategy

In this study, the databases of PubMed, Web of Science, Cochrane Library, Ebsco, China National Knowledge Infrastructure (CNKI), VIP Information Chinese Periodical Service Platform (VIP), WanFang Data, and ClinicalTrails (http://www.clinicaltrial.gov) were searched from the date of their inception to January 2024. The selection of ASCVD-related keywords was primarily based on authoritative guidelines and published literature to ensure comprehensive coverage of the topic. Advanced search functions of each database were fully utilized to refine the results. These included Boolean operators (AND, OR, NOT) and field restrictions (e.g., title, abstract, keywords). Medical Subject Headings and text search words included patients [“atherosclerosis” or “arteriosclerotic” or “cardiovascular disease” or “cardiovascular disease” or “major adverse cardiac events” or “acute coronary syndrome” or “coronary artery disease” or “coronary heart disease” or “revascularization” or “ischemic cardiomyopathy” or “stroke” or “apoplexy” or “transient ischemic attack” or “peripheral arterial disease” or “myocardial infarction” or “heart attack” or “stable angina” or “unstable angina” or “aortic aneurysm” or “cerebrovascular disease” or “cerebrovascular disorders” or “coronary artery bypass” or “percutaneous coronary intervention” or “percutaneous transluminal coronary angioplasty”] (1, 3, 36, 37) and intervention [“tanshinone IIA” or “Tan IIA” or “TNA” or “TSIIA” or “Tansh” or “sodium tanshinone IIA silate” or “sodium tanshinone IIA sulfonate injection” or “STS”]. References cited by the included studies were traced to uncover relevant additional studies. Supplementary Tables S1, S2 show the complete search strategy for the above English and Chinese databases.

### 2.2 Inclusion and exclusion criteria

Studies were included based on the PICOS criteria. (1) Type of participants (P): the study population consisted of Chinese patients diagnosed with AS or ASCVD. The ASCVD included acute coronary syndrome, stable angina, unstable angina, coronary artery disease, revascularization, ischemic cardiomyopathy, stroke, transient ischemic attack, peripheral arterial disease, etc. (2) Type of intervention (I): the experimental group was treated with STS or STS combined with other treatments; (3) Types of comparators (C): the control group was treated with non-STS treatments; (4) Types of outcome measures (O): RCTs compare outcomes in pro-inflammatory cytokines (e.g., IL-6, TNF-α, IL-1β), adhesion molecules (e.g., ICAM-1, p-selectin) and chemokines (e.g., MCP-1, fractalkine); (5) Type of studies (S): RCTs without limits on methods.

Studies that met the following criteria were excluded: (1) duplicate and non-full-text publications, including conference abstracts, bulletins, and publications that lacked complete data or methodological details; (2) reviews, non-human studies, and retrospective and observational studies; (3) published in languages other than Chinese or English.

### 2.3 Study selection and data extraction

Studies from the databases were screened and duplicates were removed using NoteExpress software. Two researchers (XYF and WBL) independently selected the studies, and conflicts between the two researchers were solved by a third researcher's opinion (FX). Eligibility screening was carried out in two steps: (1) title and abstract screening for relevance to the study objective, and (2) full-text screening for eligibility for meta-analysis.

For eligible studies, two researchers (XYF and WBL) independently extracted data from each selected RCT using a standard Excel information extraction form, and conflicts between the two researchers were solved by a third researcher's opinion (FX). The extracted information included: (1) basic information (e.g., first author, year of publication, sample size, etc.); (2) baseline characteristics of the intervention and study population (e.g., type of disease, duration of intervention, etc.); (3) relevant outcomes, including pro-inflammatory cytokines IL-6, TNF-α, and IL-1β; adhesion molecules ICAM-1 and p-selectin; chemokines fractalkine and MCP-1.

### 2.4 Risk of bias assessment

According to the Cochrane Risk of Bias Tool 2.0 (RoB2) (38), two researchers (XYF and WBL) independently assessed the risk of bias in the included trials. Five characteristics were evaluated: randomization process, deviations from the intended interventions, missing outcome data, measurement of outcomes, and selection of the reported result. Each domain was categorized as “low risk of bias”, “some risk of bias”, or “high risk of bias”. Conflicts between the two researchers were solved by the opinion of a third researcher (FX).

### 2.5 Data analysis

This study was undertaken in Review Manager software (RevMan, version 5.4, The Cochrane Collaboration, 2020) for data analysis of outcomes. Standardized mean differences (SMDs) and 95% confidence intervals (95% CIs) were used to assess continuous outcomes, with *p*-values ≤ 0.05 considered to be statistically significant.

#### 2.5.1 Heterogeneity assessment

Heterogeneity among the included studies was assessed using I-squared (*I*^2^) and *p* values of the Chi-square test. *I*^2^ were interpreted as follows: 0%–25% (low heterogeneity), 26%–50% (moderate heterogeneity), and >50% (substantial heterogeneity). A *p*-value ≥ 0.10 or *I*^2^ ≤ 50% was considered indicative of no significant heterogeneity.

#### 2.5.2 Model selection

According to the results of the heterogeneity test, either a random-effects model or a fixed-effects model was selected for data analysis. When there was no statistical heterogeneity (*p*-value ≥ 0.10, or *I^2^* ≤ 50%), a fixed-effects model was selected; otherwise, a random-effects model was used.

#### 2.5.3 Sensitivity and subgroup analyses

When significant heterogeneity was identified, its source was further evaluated by sensitivity analyses or subgroup analyses. Sensitivity analyses were conducted to assess the effect of each trial on the validity of the pooled overall SMDs using the leave-one-out method. Subgroup analyses were conducted to explore potential sources of heterogeneity and assess variations in effect sizes. Subgroups were categorized based on the following variables: type of conditions [CAD vs. unstable angina vs. acute myocardial infarction (AMI) vs. acute coronary syndrome (ACS) vs. ischemic stroke vs. AS combined with other indications], dosage of STS (40 mg/d vs. 50 mg/d vs. 60 mg/d vs. 80 mg/d vs. unclear), and duration of treatment (1 week vs. 2 weeks vs. 3 weeks vs. 4 weeks vs. 7 weeks vs. 12 weeks), and each subgroup should contain at least 3 studies to ensure the validity and reliability of the subgroup analyses (39).

#### 2.5.4 Publication bias assessment

The results of the meta-analysis were presented in forest plots, and publication bias was detected by the funnel plot symmetry test and Egger's regression test. Egger's regression test was undertaken in Stata (Version 17.0; Stata Corp, College Station, TX) when 10 or more studies were included.

## 3 Results

### 3.1 Search results

**Figure 1:**
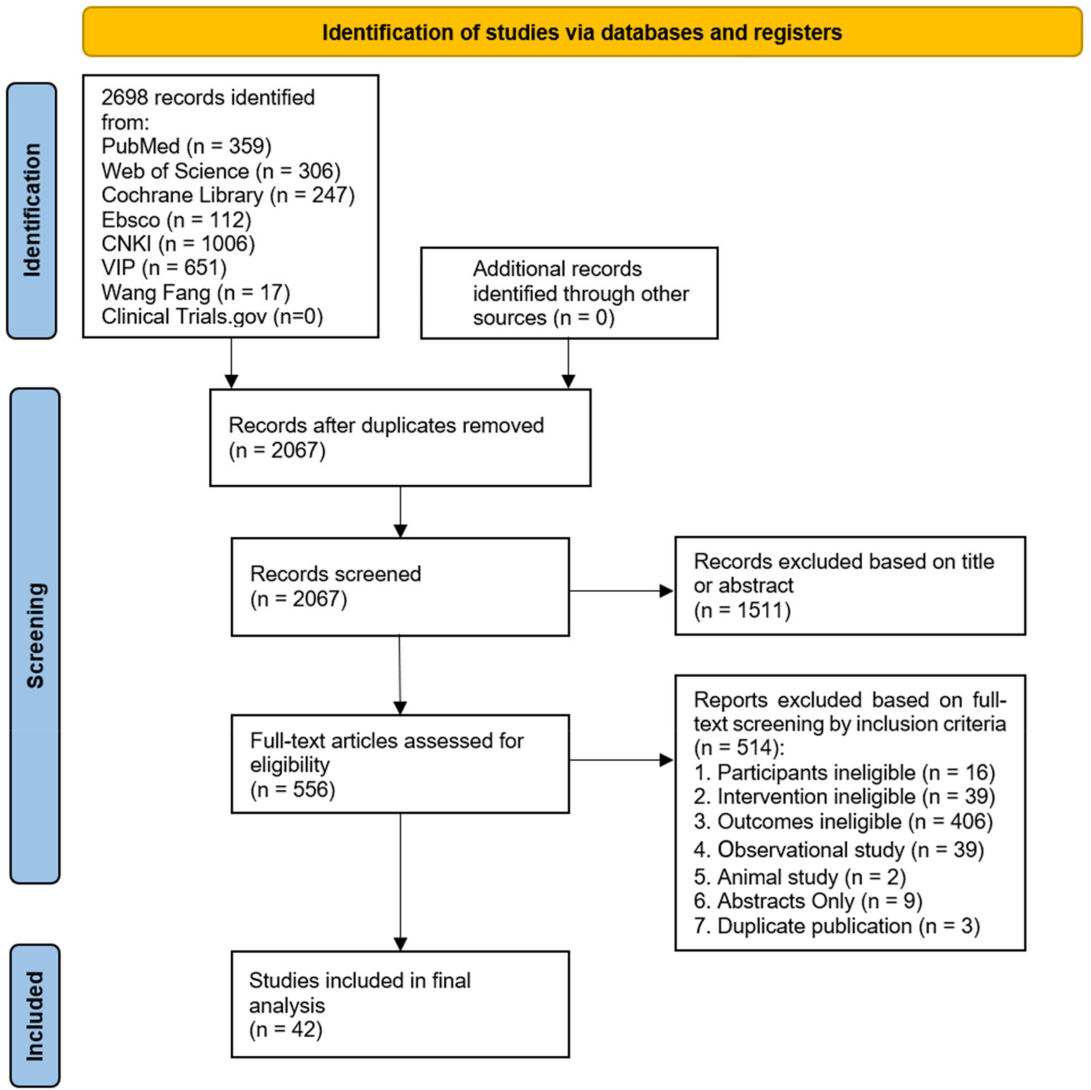
Flowchart of the search, inclusion, and exclusion study selection.

The initial search yielded 2,698 articles, and after deleting duplicates, 2,067 articles were initially screened for title and abstract. 1,511 articles were subsequently excluded, 556 articles were potentially eligible, and these were retrieved for full-text review. After reading the full text, 514 articles were excluded because they failed to meet the inclusion criteria. Ultimately, 42 studies that fully met the pre-established inclusion criteria of this meta-analysis were included. The search procedure and reasons for exclusion are shown in Figure 1.

### 3.2 Study characteristics

The 42 included studies were published between 2008 and 2023 (32, 33, 40–79), with a total of 2,330 individuals in the experimental group and 2,324 in the control group. There were 26 studies that reported the level of IL-6, 25 that reported the level of TNF-α, 2 that reported the level of IL-1β, 3 that reported the level of ICAM-1 levels, 3 that reported the level of p-selectin, 3 that reported the level of fractalkine, and 3 that reported the level of MCP-1. The main characteristics of these studies are presented in Table 1.

**Table 1:** Characteristics of included studies.

Study	Population	Sample size (E/C)	Age		Interventions		Duration	Outcomes
			E	C	E	C		
(40)	CAD	49/49	61.49 ± 9.14	62.59 ± 8.21	CTs + STS 60 mg/d	CTs	4 weeks	TNF-α, ICAM-1
(41)	AMI	40/40	60.5 ± 2.5	61.5 ± 2.0	Reteplase + Aspirin + LMWH + STS 60 mg/d	Reteplase + Aspirin + LMWH	1 week	IL-6, TNF-α
(42)	Ischemic stroke	33/34	74.8 ± 8.6	75.7 ± 7.8	CTs + STS 60 mg/d	CTs + PGI 500 mg/d	2 weeks	IL-6
(43)	CAD	42/42	65.4 ± 3.2	64.5 ± 4.1	CTs + STS 40 ∼ 80 mg/d	CTs	2 weeks	IL–6, TNF-α
(44)	CAD	43/43	58.25 ± 8.17	60.24 ± 8.45	STS 60 mg/d	Isosorbide dinitrate 40 mg/d	2 weeks	IL-6, TNF-α
(45)	CAD	53/53	64.09 ± 3.28	64.04 ± 3.53	Atorvastatin + STS 60 ∼ 80 mg/d	Atorvastatin 20 mg/d	3 weeks	IL-6, TNF-α
(46)	ACS	40/40	N/A	N/A	CTs + STS 80 mg/d	CTs	2 weeks	TNF-α
(47)	CAD	100/100	61.98 ± 6.34	61.76 ± 5.98	Atorvastatin + STS 80 mg/d	Atorvastatin 20 mg/d	4 weeks	IL-6, TNF-α
(48)	AMI	47/42	59.7 ± 5.6	58.9 ± 4.4	CTs + STS 60 mg/d	CTs	1 week	TNF-α
(49)	ACS	61/60	59.6 ± 14.1	58.9 ± 13.6	CTs + STS 50 mg/d	CTs	2 weeks	TNF-α
(50)	CAD	80/80	62.5 ± 8.5	62.7 ± 8.7	Atorvastatin + STS 80 mg/d	Atorvastatin 10 mg/d	4 weeks	IL-6, TNF-α, ICAM-1
(51)	CAD	42/42	59.37 ± 8.25	61.10 ± 8.78	CTs + STS 60 mg/d	CTs	2 weeks	TNF-α
(52)	Unstable angina	65/65	61.5 ± 8.6	62.6 ± 9.3	CTs + STS 60 mg/d	CTs	1 week	p-selectin
(53)	Ischemic stroke	64/64	68.7 ± 7.1	69.1 ± 6.7	CTs + STS 60 mg/d	CTs	2 weeks	IL-6, IL-1β
(54)	ACS	61/60	59.6 ± 14.1	58.9 ± 13.6	CTs + STS 50 mg/d	CTs	2 weeks	TNF-α
(32)	CAD	35/35	66 ± 7.28	67.47 ± 6.52	Atorvastatin + STS 80 mg/d	Atorvastatin 20 mg/d	2 weeks	IL-6, TNF-α
(55)	AMI	35/35	61.3 ± 4.8	61.5 ± 4.4	CTs + STS 60 mg/d	CTs	1 week	fractalkine
(56)	ACS	49/49	55.98 ± 5.49	56.44 ± 5.86	CTs + STS 50 mg/d	CTs	2 weeks	IL-6, TNF-α, MCP-1
(57)	CAD	51/51	57.51 ± 3.26	56.38 ± 3.57	CTs + Trimetazidine Hydrochloride + STS 40 ∼ 80 mg/d	CTs + Trimetazidine Hydrochloride 60 mg/d	8 weeks	IL-6, TNF-α
(58)	Ischemic stroke	110/110	64.37 ± 2.30	65.40 ± 1.20	CTs + STS 60 mg/d	CTs	4 weeks	Fractalkine
(59)	AMI	53/53	71.43 ± 6.12	70.51 ± 6.09	Aspirin + Atorvastatin + Trimetazidine Hydrochloride + Urokinase + STS 40 mg/d	Aspirin + Atorvastatin + Trimetazidine Hydrochloride 60 mg/d + Urokinase 4,000 U/kg/d	2 weeks	Fractalkine
(60)	CAD	55/55	65.17 ± 8.34	64.82 ± 7.61	Bisoprolol fumarate + STS 60 mg/d	Bisoprolol fumarate 5 mg/d	4 weeks	IL-6, MCP-1
(61)	ACS	59/59	70.89 ± 7.18	71.21 ± 7.21	CTs + STS 80 mg/d	CTs	2 weeks	TNF-α
(62)	CAD	32/32	57.03 ± 3.95	56.38 ± 3.72	Atorvastatin + STS 60 ∼ 80 mg/d	Atorvastatin 20 mg/d	3 weeks	TNF-α
(63)	CAD	55/55	61.50 ± 7.30	61.30 ± 8.50	Atorvastatin + STS 80 mg/d	Atorvastatin 20 mg/d	4 weeks	IL-6, TNF-α
(64)	ACS	40/40	58.7 ± 7.8	58.0 ± 6.9	CTs + STS 40 mg/d	CTs	7 weeks	IL-6
(65)	CAD combined with AS	75/75	63.22 ± 7.45	63.16 ± 7.49	Rosuvastatin + STS 80 mg/d	Rosuvastatin 20 mg/d	12 weeks	IL-6
(66)	CAD	30/30	N/A	N/A	CTs + STS 80 mg/d	CTs	2 weeks	TNF-α
(67)	CAD	80/80	60 ± 9	59 ± 9	Atorvastatin + STS 80 mg/d	Atorvastatin 10 mg/d	4 weeks	IL-6
(68)	Unstable angina	31/31	59.20 ± 11.12	60.04 ± 10.23	CTs + STS 60 mg/d	CTs	4 weeks	TNF-α
(69)	CAD	41/41	62.4 ± 2.5	62.5 ± 2.8	CTs + Aspirin + Metoprolol tartrate + STS 60 mg/d	CTs + Aspirin 100 mg/d + Metoprolol tartrate 100 mg/d	4 weeks	p-selectin
(70)	Ischemic stroke	57/57	64.8 ± 6.2	64.5 ± 6.0	CTs + STS 40 mg/d	CTs	2 weeks	IL-6, p-selectin
(71)	Unstable angina	37/37	65.8 ± 8.2	62.5 ± 6.9	CTs + STS 80 mg/d	CTs	1 week	IL-6, TNF-α
(33)	CAD	80/80	60.12 ± 9.47	58.75 ± 9.25	Atorvastatin + STS 80 mg/d	Atorvastatin 10 mg/d	4 weeks	IL-6
(72)	unstable angina	32/32	59 ± 11	60 ± 10	CTs + STS 60 mg/d	CTs	1 week	TNF-α, MCP-1
(73)	unstable angina	40/40	63.5 ± 12.3	66.3 ± 9.2	CTs + STS 0.06 mg/d	CTs	2 weeks	TNF-α
(74)	CAD	105/105	60.3 ± 4.1	59.2 ± 4.8	Simvastatin + STS 60 mg/d	Simvastatin 10 mg/d	4 weeks	IL-6, TNF-α
(75)	AMI	102/102/102	56 ± 5.3	54 ± 6.3	Clopidogrel + Aspirin + LMWH + STS 50 mg/d	Clopidogrel + Aspirin + LMWH	1 week	TNF-α
(76)	AMI	74/74	56.28 ± 7.43	56.73 ± 7.85	Amiodarone hydrochloride + STS 40 mg/d	Amiodarone hydrochloride < 1,200 mg/d	2 weeks	IL-6, TNF-α, IL-1β, ICAM-1
(77)	T2DM combined with AS	45/45	65.01 ± 5.02	64.21 ± 4.73	CTs + STS 80 mg/d	CTs	2 weeks	IL-6
(78)	CAD	47/47	52.71 ± 5.42	52.46 ± 5.38	Clopidogrel + Aspirin + STS 80 mg/d	Clopidogrel 75 mg/d + Aspirin 100 mg/d	2 weeks	IL-6
(79)	Ischemic stroke	60/60	62.06 ± 13.92	61.24 ± 11.69	Aspirin + STS 80 mg/d	Aspirin 100 mg/d	3 weeks	TNF-α, ICAM-1

ACS, acute coronary syndrome; AMI, acute myocardial infarction; AS, atherosclerosis; C, control group; CAD, coronary artery disease; CTs, conventional therapy, such as anticoagulant therapy, antiplatelet agents, nitrates, statins, β-blockers, angiotensin converting enzyme inhibitors, angiotensin receptor blockers, calcium channel blockers, etc. Different patients used different drugs for conventional therapy. E, experimental group; ICAM-1, intercellular adhesion molecule-1; IL-1β, interleukin-1β; IL-6, interleukin-6; LMWH, low molecular weight heparin sodium; MCP-1, monocyte chemoattractant protein-1; N/A, the date was not reported; PGI, puerarin and glucose injection; STS, sodium tanshinone IIA sulfonate; T2DM, type 2 diabetes mellitus; TNF-α, tumor necrosis factor-α; w, weeks; X mg/d, X mg daily.

### 3.3 Risk of bias assessment

For “bias arising from the randomization process”, 42 trials mentioned randomization, and 10 trials (40–42, 44, 46, 51, 52, 57, 62, 63) explicitly mentioned the fact that the order of allocation was randomized by random number tables or lotteries, which was therefore considered to be “low risk”.

For “bias due to deviations from intended interventions”, only two trials (32, 42) used blinding for participants and operators, and three trials (66, 71, 79) were rated as “high risk” because they did not use blinding for participants and operators and did not mention whether appropriate analyses were used to estimate the effects of assignment to intervention.

For “bias due to missing outcome data”, five trials were rated as “high risk”, including four trials (40–42, 64) lacking specific information and one trial (42) failing to explain the reason for missing follow-up.

For “bias in measurement of the outcome”, four trials (40–42, 64) were rated as “low risk”, one (42) of which used blinding for the assessor, and the remaining three trials had no impact on the judgment of outcome despite the assessor's knowledge of the intervention.

For “bias in selection of the reported result”, all trials were rated as “low risk”, and data from all trials were analyzed in accordance with a prespecified analysis plan, with no artificial selection of results.

**Figure 2:**
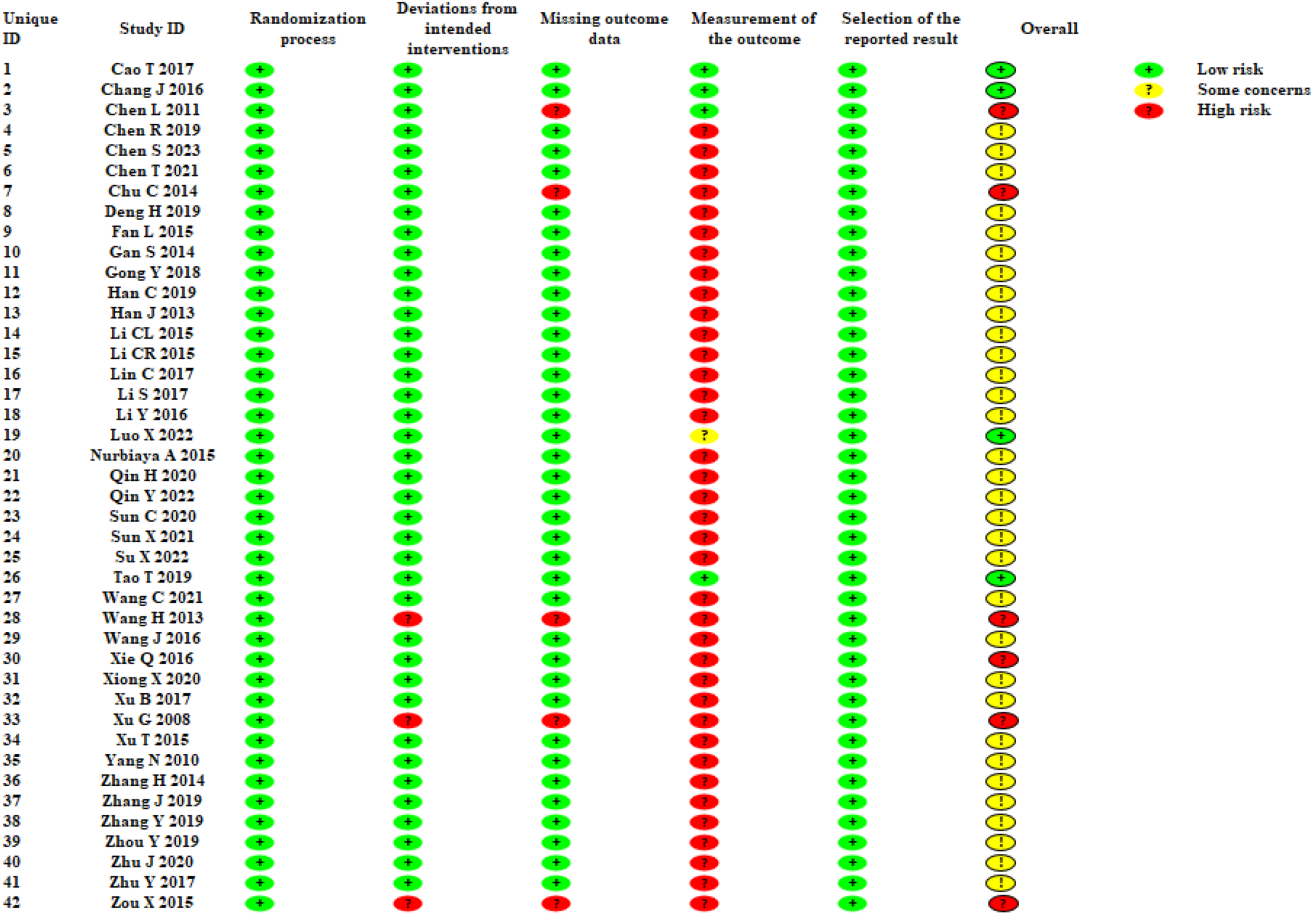
Bias risk assessment of included studies.

Based on the above five aspects of bias risk assessment, the overall bias risk of the trials was derived. Four trials (40, 41, 57, 64) were rated as “low risk”, six trials (42, 46, 66, 68, 71, 79) were assessed as “high risk”, and the other trials were assessed as “some risk”. Specific information on the bias risk assessment is shown in Figure 2. Supplementary Figure S1 shows the overall risk of bias.

### 3.4 Main outcomes

#### 3.4.1 Pro-inflammatory cytokines

**Figure 3:**
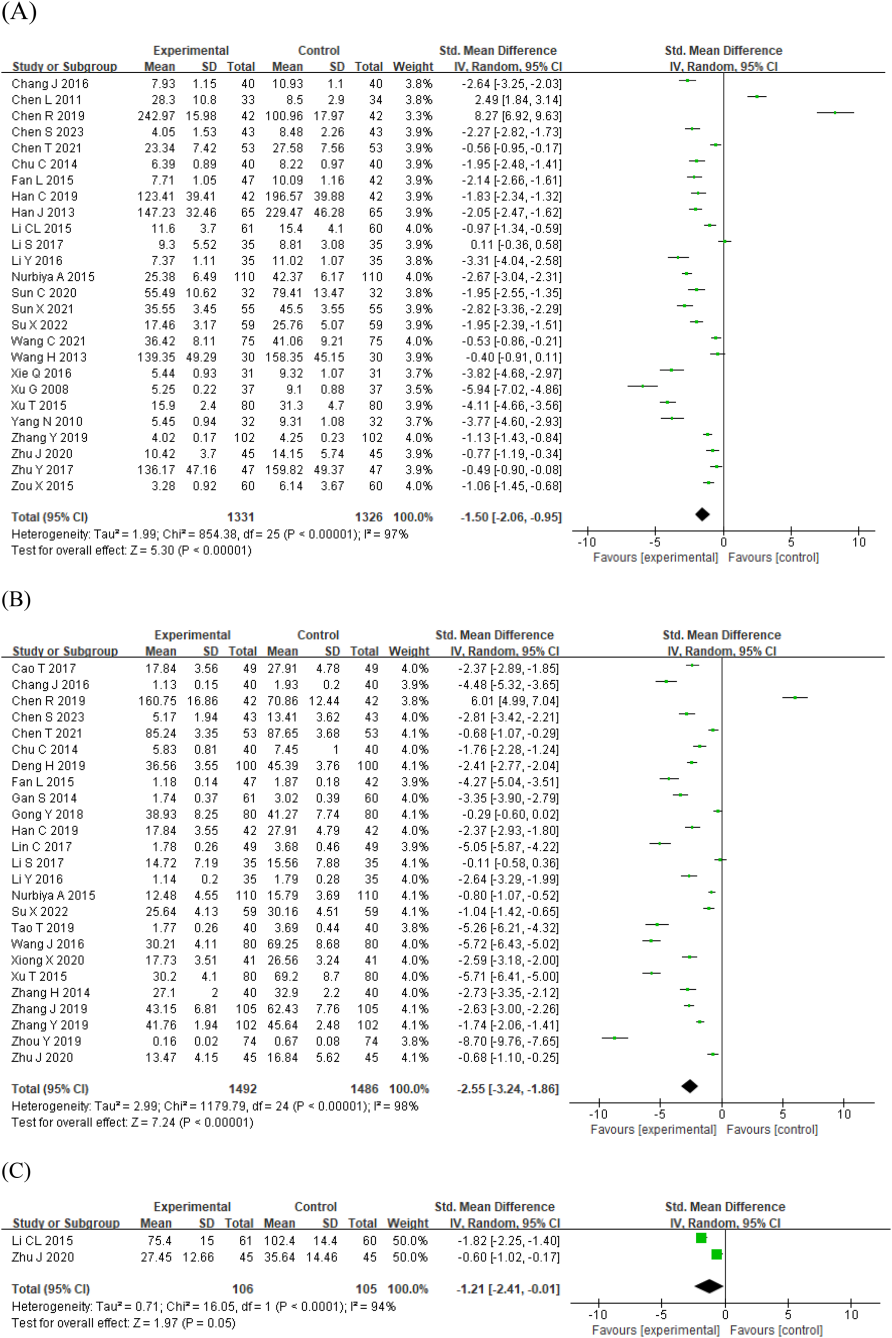
Forest plots of the effect of STS on pro-inflammatory cytokines: **(A)** IL-6 **(B)** TNF-α **(C)** IL-1β. IL-1β, interleukin-1β; IL-6, interleukin-6; STS, sodium tanshinone IIA sulfonate; TNF-α, tumor necrosis factor-α.

The three pro-inflammatory cytokines IL-6, TNF-α, and IL-1β showed a high degree of heterogeneity among the studies, so the random effects model was used. The pooled findings showed that using STS in patients with AS and ASCVD significantly decreased the level of IL-6 [SMD = −1.50, 95% CI (−2.06, −0.95), *p* < 0.00001; *I*^2^ = 97%; Figure 3A], TNF-α [SMD = −2.55, 95% CI (−3.24, −1.86), *p* < 0.00001; *I*^2^ = 98%; Figure 3B], and IL-1β [SMD = −1.21, 95% CI (−2.41, −0.01), *p* < 0.0001; *I*^2^ = 94%; Figure 3C].

#### 3.4.2 Adhesion molecules

The adhesion molecule ICAM-1 did not show a high degree of heterogeneity among the studies, so a fixed-effects model was used, and the pooled findings showed that STS significantly decreased the level of ICAM-1 [SMD = −1.28, 95% CI (−1.55, −1.02), *p* < 0.00001; *I*^2^ = 0%; Supplementary Figure S2A]. The adhesion molecule p-selectin was used in a random-effects model, and the pooled results showed that STS significantly decreased the level of p-selectin [SMD = −1.06, 95% CI (−1.46, −0.67), *p* < 0.00001; *I*^2^ = 63%; Supplementary Figure S2B].

#### 3.4.3 Chemokines

A random-effects model was used for the chemokine fractalkine, and the pooled results showed that STS significantly decreased the level of fractalkine [SMD = −1.32, 95% CI (−2.02, −0.61), *p* = 0.0003; *I*^2^ = 88%; Supplementary Figure S3]. A fixed-effects model was used for the chemokine MCP-1, and the pooled results showed that STS significantly decreased the level of MCP-1 [SMD = −0.83, 95% CI (−1.11, −0.55), *p* < 0.00001; *I*^2^ = 0%; Supplementary Figure S3B].

### 3.5 Heterogeneity

#### 3.5.1 Sensitivity analyses

Meta-analysis showed statistically significant heterogeneity for the outcomes of IL-6 (*I*^2^ = 97%, *p* < 0.10), TNF-α (*I*^2^ = 98%, *p* < 0.10), IL-1β (*I*^2^ = 94%, *p* < 0.10), p-selectin (*I*^2^ = 63%, *p* < 0.10) and fractalkine (*I*^2^ = 88%, *p* < 0.10). Since only 2 articles were included for IL-1β, and arbitrary exclusion of either one would result in unmeasured heterogeneity, sensitivity analyses were performed only for the remaining 4 outcomes. Heterogeneity did not change significantly after the exclusion of individual studies. There was not any statistically significant difference before or after sensitivity pooled SMDs for IL-6, TNF-α, p-selectin, and fractalkine concentration levels, as shown in Table 2.

**Table 2:** Sensitivity analyses of STS's influence on IL-6, TNF-α, p-selectin, and fractalkine.

Outcomes	Pre-sensitivity analyses			Upper and lower of effect size	Post-sensitivity analyses		
	No. of studies included	Pooled SMD (RE)	95%CI		Pooled SMD (RE)	95%CI	Excluded studies
IL-6	26	−1.5	[−2.06, −0.95]	Upper	−1.35	[−1.89, −0.80]	(71)
				Lower	−1.82	[−2.32, −1.33]	(43)
TNF-α	25	−2.55	[−3.24, −1.86]	Upper	−2.31	[−2.96, −1.65]	(76)
				Lower	−2.88	[−3.51, −2.24]	(43)
p-selectin	3	−1.06	[−1.46, −0.67]	Upper	−0.90	[−1.16, −0.63]	(71)
				Lower	−1.1	[−1.84, −0.35]	(53)
fractalkine	3	−1.32	[−2.02, −0.61]	Upper	−0.96	[−1.24, −0.67]	(57)
				Lower	−1.42	[−2.67, −0.17]	(60)

IL−6, interleukin−6; RE, random effect; SMD, standardized mean differences; STS, sodium tanshinone; IIA, sulfonate; TNF-α, tumor necrosis factor-alpha.

#### 3.5.2 Subgroup analyses

As there were only 2–3 included studies for the other outcomes, this study conducted subgroup analyses of IL-6 and TNF-α. Following subgroup analyses, heterogeneity was changed among some of the strata of subgroups. There were significant differences before or after subgroup analyses in the stratum of coronary artery disease [SMD = −0.70, 95% CI (−1.82, 0.42), *p* > 0.05] and the stratum of ischaemic stroke [SMD = −0.43, 95% CI (−2.91, 2.05), *p* > 0.05] for IL-6. There were significant differences before or after subgroup analyses in the stratum of the group whose STS dose was unspecified [SMD = 1.84, 95% CI (−1.78, 5.46), *p* > 0.05] for IL-6. There were significant differences before or after subgroup analyses in the stratum of the group whose STS dose was unspecified [SMD = 2.65, 95% CI (−3.91, 9.21), *p* > 0.05] for TNF-α. These results of subgroup analyses suggested that the type of conditions and dosage of STS may be the source of heterogeneity in the meta-analysis. Supplementary Table S3 shows the subgroup analysis of the effects of STS on IL-6 and TNF-α.

### 3.6 Publication bias

**Figure 4:**
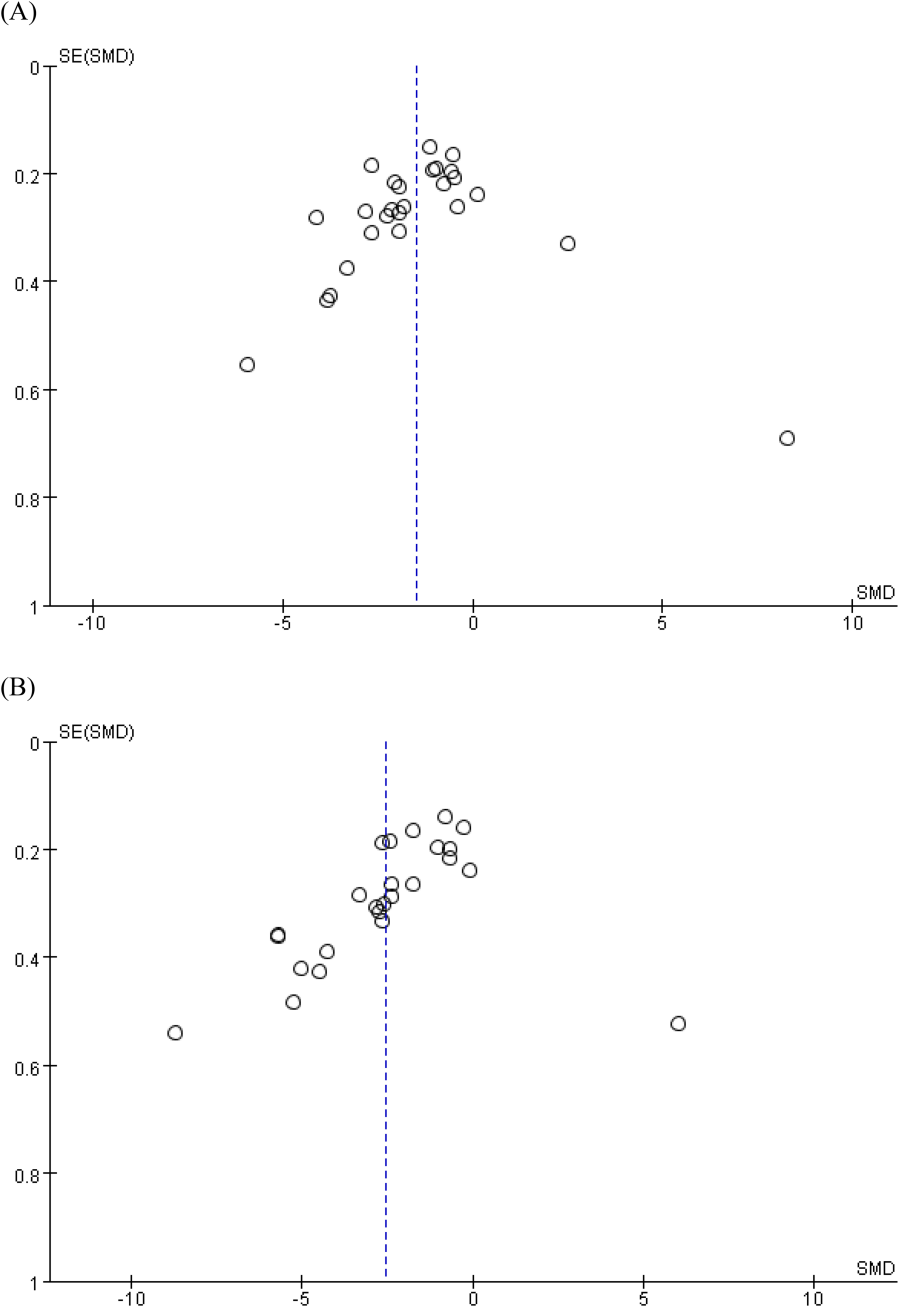
Funnel charts based on **(A)** IL-6 and **(B)** TNF-α. IL-6, interleukin-6; TNF-α, tumor necrosis factor-alpha.

Due to there being fewer than 10 included studies for the other outcomes, only the levels of IL-6 and TNF-α were chosen to perform the funnel plot and Egger's regression test in this study. These tests showed no significant evidence of publication bias for meta-analysis assessing the effect of STS on IL-6 [*p* = 0.447, 95% CI (−11.07, 5.04)] concentration levels. However, as shown in Figure 4, the asymmetry displayed in the funnel plot, and Egger's regression test [*p* = 0.004, 95% CI (−17.97, −3.74)] of TNF-α indicated that there might be some publication bias, which might be because the positive results were selectively published.

## 4 Discussion

Salvia miltiorrhiza is a kind of traditional Chinese medicine with a long history, and its fat-soluble active component, Tan IIA, has been widely used in the treatment of a variety of diseases. STS, as the most widely used clinical preparation of Tan IIA in China, has a long history of clinical application and a large number of human efficacy trials in the field of AS and ASCVD. Many studies have been conducted to systematically evaluate the effects of STS in the treatment of diseases such as coronary artery disease, unstable angina, and acute cerebral infarction, mainly investigating the effectiveness, safety, and effects on blood lipids (80–82). In contrast, there are fewer systematic reviews and meta-analyses of clinical trials on the anti-inflammatory effects of STS. Kan et al. (34), and Yu et al. (35) have only studied one disease in ASCVD, which could not be directly derived to the effects on a class of diseases. Meanwhile, existing studies have focused more on the effects of STS on pro-inflammatory cytokines, such as IL-6 and TNF-α, while neglecting its effects on adhesion molecules and chemokines, which also play an important role in the inflammatory process of atherosclerosis. Therefore, this study provides reliable evidence that STS exerts anti-inflammatory effects to treat AS and ASCVD through influencing pro-inflammatory cytokines, adhesion molecules, and chemokines, which can help guide clinical decision-making.

The meta-analysis included 42 RCTs and involved 4,654 Chinese patients with AS and ASCVD, assessing the effects of STS on pro-inflammatory cytokines, adhesion molecules, and chemokines. The results suggested that STS could significantly reduce the concentration levels of pro-inflammatory cytokines IL-6, TNF-α, and IL-1β, adhesion molecules ICAM-1 and p-selectin, and chemokines fractalkine and MCP-1 in AS and ASCVD patients.

The significant reduction in pro-inflammatory cytokines observed in this study suggests that STS may have beneficial effects on cardiovascular events. IL-6, TNF-α, and IL-1β are key mediators of chronic inflammation. IL-1β, in particular, has been shown to activate inflammatory pathways, leading to immune cell recruitment at the site of vascular injury and the formation of unstable plaques. These cytokines play a crucial role in the progression of atherosclerosis and the destabilization of plaques, both of which are major contributors to cardiovascular events such as myocardial infarction and stroke (10, 30). Reducing these cytokines may help stabilize plaques and alleviate endothelial dysfunction, thereby reducing the incidence of acute cardiovascular events (83). Additionally, the reduction in adhesion molecules (ICAM-1 and p-selectin) and chemokines (fractalkine and MCP-1) indicates an attenuation of leukocyte recruitment and adhesion, which are essential steps in the formation of atherosclerotic plaques (84, 85). By modulating these pathways, STS may help mitigate the inflammatory response and vascular injury, offering a potential mechanism for improving long-term cardiovascular outcomes.

This study explored heterogeneity and its impact on meta-analysis results through sensitivity and subgroup analyses. Despite significant heterogeneity for IL-6, TNF-α, IL-1β, p-selectin, and fractalkine, sensitivity analyses showed that excluding individual studies did not significantly affect heterogeneity or pooled effect sizes, suggesting the robustness of the meta-analysis results. Subgroup analyses identified the type of conditions and the dosage of STS as key sources of heterogeneity. Significant changes in the levels of IL-6 and TNF-α before and after subgroup analyses underscore the varying effects of disease state and treatment dose on the efficacy of STS. These findings highlight the importance of considering disease heterogeneity and treatment dosage in clinical trial design. Tailored interventions based on disease type and STS dosage may be more effective in improving cardiovascular outcomes in ASCVD patients.

However, some potential sources of heterogeneity were not analyzed due to limited data. For example, differences in the manufacturers of STS could influence the results due to variations in product quality and formulation. Additionally, the duration of disease progression may affect how patients respond to treatment, with those in the earlier stages potentially benefiting more from the intervention. While subgroup analyses for these factors were not conducted, they remain important considerations for future research. Furthermore, differences in assay methods, such as ELISA and radioimmunoassay, may introduce variability due to their distinct sensitivities and specificities. Although these factors were not specifically addressed in this analysis, they represent areas for further investigation to better understand their impact on treatment outcomes in future studies.

In this study, funnel plots and Egger's regression tests were employed to assess potential publication bias. For IL-6, neither the funnel plot nor Egger's regression test provided substantial evidence of publication bias. However, for TNF-α, Egger's regression test revealed significant publication bias, and the funnel plot showed noticeable asymmetry. This asymmetry suggests that positive results may have been selectively published, while negative or insignificant findings might not have been reported. Such selective publication could lead to an overestimation of the effect of STS on TNF-α, potentially influencing our overall assessment of its efficacy. To mitigate the impact of publication bias, we recommend that future studies conduct a more comprehensive literature search strategy to include all relevant studies, regardless of their findings. Researchers should also consider using registration protocols to prespecify study designs, analysis plans, and outcome reporting, which can help minimize selective reporting. Finally, journals should encourage transparency and integrity by requiring authors to report all prespecified outcomes, including those that are not statistically significant.

The meta-analysis by Kan et al. (34) showed that there was insufficient evidence that STS inhibited the expression of IL-6, which differs from our findings that STS significantly reduced the level of IL-6. Our findings are consistent with the conclusions of the systematic review by Yu et al. (35). The differences in the findings may be due to the differences in patient populations. The study by Kan et al. focused on patients with ACS after PCI, and the study by Yu et al. focused on patients with coronary artery disease. In contrast, the results of the present study were derived based on a wider range of patient populations with AS and ASCVD, to explore the overall impact of STS on this large group. Furthermore, in the subgroup analysis, it can be concluded that STS significantly reduced the level of IL-6 [SMD = −1.61, 95%CI (−2.30, −0.91), *p* < 0.00001; *I*^2^ = 86%] in patients with ACS, since the subgroup analysis only made a distinction in terms of types of disease but did not limit to patients with ACS after PCI. The treatment of ACS depends on several factors, including the severity of the disease, the overall health of patients, and the time duration since the onset of the disease. PCI is one of the common methods of treating ACS but is not suitable for all patients. Taken together with the results of the subgroup analyses, STS did not have a significant effect on lowering the levels of IL-6 for patients with coronary artery disease and ischemic stroke, but it had a significant effect on patients who suffer from unstable angina and acute myocardial infarction. This reveals that STS may have specific effects on patients with different disease states, facilitating the development of individualized treatment plans.

There are a few limitations to this meta-analysis that should be acknowledged. Firstly, a preliminary search revealed that all the relevant RCTs included in this study were conducted exclusively in Chinese populations. This limits the generalizability of the findings to other ethnic groups or geographic regions, as genetic, environmental, and cultural factors may influence the effects of STS. To address this limitation, future studies should aim to include diverse populations, and efforts should be made to explore the applicability of the findings to different ethnic groups and regions. Secondly, the number of RCTs for IL-1β, ICAM-1, p-selectin, fractalkine, and MCP-1 is relatively small. This may introduce uncertainty in the results, particularly for these markers. To enhance the robustness of the evidence, larger clinical trials targeting these specific biomarkers are needed. Additionally, the inclusion of grey literature, such as unpublished data and conference abstracts, could help provide a more comprehensive understanding of STS's effects across various biomarkers. Thirdly, there is an inherent risk of bias in the included studies. Blinding the participants, operators, and assessors is difficult to achieve because the STS solution is unique in color and difficult to mimic, and most of the controls in the included trials were oral-type interventions. Potential differences in methods of random sequence generation and allocation concealment in the included studies may lead to a high degree of heterogeneity in the results of the meta-analysis. To minimize bias, care should be taken to protect information about patients’ treatment allocation, and all study participants should be discouraged from discussing their participation in the trial, reducing interaction between patient groups. Finally, significant heterogeneity and publication bias were observed in the results, which necessitate cautious interpretation of the sources and outcomes. Future research should aim to reduce publication bias by including all relevant studies, regardless of their results, and by adopting more transparent and standardized reporting practices.

## 5 Conclusion

The use of STS in patients with AS and ASCVD appears to have significantly decreased the levels of pro-inflammatory cytokines IL-6, TNF-α, and IL-1β, adhesion molecules ICAM-1 and p-selectin, and chemokines fractalkine and MCP-1. This study provides new and useful evidence to support clinical medication decisions for AS and ASCVD (85–87).

## Data Availability

The datasets presented in this study can be found in online repositories. The names of the repository/repositories and accession number(s) can be found in the article/Supplementary Material.
