# Supplementary material for: Effects of sodium tanshinone IIA sulfonate injection on pro-inflammatory cytokines, adhesion molecules and chemokines in Chinese patients with atherosclerosis and atherosclerotic cardiovascular disease: a meta-analysis of randomized controlled trials
Source: Front Cardiovasc Med. 2025 Feb 13;12:1511747. doi: 10.3389/fcvm.2025.1511747 (PMC11865200; doi:10.3389/fcvm.2025.1511747)
Supplement: Supplementary file 1 [file Datasheet1.pdf]

Supplementary Material

1 Supplementary Tables and Figures

1.1 Supplementary Tables

1.1.1 Supplementary Table 1: The search strategy for English databases.

Database	Order	Strategy
PubMed	#1	"Atherosclerosis"[MeSH Terms] OR "Cardiovascular Diseases"[MeSH Terms] OR "Acute Coronary Syndrome"[MeSH Terms] OR "Coronary Artery Disease"[MeSH Terms] OR "Coronary Disease"[MeSH Terms] OR "Cardiomyopathies"[MeSH Terms] OR "Stroke"[MeSH Terms] OR "ischemic attack, transient"[MeSH Terms] OR "Peripheral Arterial Disease"[MeSH Terms] OR "Myocardial Infarction"[MeSH Terms] OR "angina, stable"[MeSH Terms] OR "angina, unstable"[MeSH Terms] OR "Aortic Aneurysm"[MeSH Terms] OR "Cerebrovascular Disorders"[MeSH Terms] OR "Coronary Artery Bypass"[MeSH Terms] OR "Percutaneous Coronary Intervention"[MeSH Terms] OR "angioplasty, balloon, coronary"[MeSH Terms]
	#2	"atheroscleros*"[Title/Abstract] OR "Atheroscleroses"[Title/Abstract] OR "arteriosclerotic cardiovascular disease"[Title/Abstract] OR "cardiovascular disease"[Title/Abstract] OR "major adverse cardiac events"[Title/Abstract] OR "acute coronary syndrome*"[Title/Abstract] OR "coronary artery disease"[Title/Abstract] OR "coronary heart disease"[Title/Abstract] OR "revascularization"[Title/Abstract] OR "ischemic cardiomyopathy"[Title/Abstract] OR "stroke*"[Title/Abstract] OR "Apoplexy"[Title/Abstract] OR "transient ischemic attack*"[Title/Abstract] OR "peripheral arterial disease*"[Title/Abstract] OR "myocardial infarct*"[Title/Abstract] OR "heart attack*"[Title/Abstract] OR "stable angina*"[Title/Abstract] OR "unstable angina*"[Title/Abstract] OR "aortic aneurysm*"[Title/Abstract] OR "cerebrovascular disease"[Title/Abstract] OR "cerebrovascular disorders"[Title/Abstract] OR "coronary artery bypass*"[Title/Abstract] OR "aortocoronary bypass*"[Title/Abstract] OR "percutaneous coronary

		intervention*"[Title/Abstract] OR "percutaneous coronary revascularization*"[Title/Abstract] OR "transluminal coronary balloon dilation"[Title/Abstract] OR "coronary balloon angioplast*"[Title/Abstract] OR "percutaneous transluminal coronary angioplast*"[Title/Abstract]
	#3	"tanshinone"[Supplementary Concept] OR "tanshinone II A sodium sulfonate"[Supplementary Concept] OR "tanshinone iia"[Title/Abstract] OR "tan iia"[Title/Abstract] OR "TNA"[Title/Abstract] OR "TSIIA"[Title/Abstract] OR "Tansh"[Title/Abstract] OR "sodium tanshinone iia silate"[Title/Abstract] OR "STS"[Title/Abstract] OR "sodium tanshinone iia sulfonate injection"[Title/Abstract] OR "tanshione"[Title/Abstract]
	#4	"randomized controlled trial"[Publication Type] OR "clinical trial"[Publication Type] OR "controlled clinical trial"[Publication Type] OR "Randomized Controlled Trials as Topic"[MeSH Terms] OR "Clinical Trials as Topic"[MeSH Terms] OR "Controlled Clinical Trials as Topic"[MeSH Terms] OR "Random Allocation"[MeSH Terms] OR "Double-Blind Method"[MeSH Terms] OR "clinical trial"[Title/Abstract] OR "clinical stud*"[Title/Abstract] OR "random*"[Title/Abstract] OR "RCT"[Title/Abstract]
	#5	(#1 OR #2) AND #3 AND #4
Web of Science	#1	TS=("atheroscleros*" OR "Atheroscleroses" OR "arteriosclerotic cardiovascular disease" OR "cardiovascular disease" OR "major adverse cardiac events" OR "acute coronary syndrome*" OR "coronary artery disease" OR "coronary heart disease" OR "revascularization" OR "ischemic cardiomyopathy" OR "stroke*" OR "Apoplexy" OR "transient ischemic attack*" OR "peripheral arterial disease*" OR "myocardial infarct*" OR "heart attack*" OR "stable angina*" OR "unstable angina*" OR "aortic aneurysm*" OR "cerebrovascular disease" OR "cerebrovascular disorders" OR "coronary artery bypass*" OR "aortocoronary bypass*" OR "percutaneous coronary intervention*" OR "percutaneous coronary revascularization*" OR "transluminal coronary balloon dilation" OR "coronary balloon angioplast*" OR "percutaneous transluminal coronary angioplast*")
	#2	TS=("tanshinone iia" OR "tan iia" OR "TNA" OR "TSIIA" OR "Tansh" OR "sodium tanshinone iia silate" OR "STS" OR "sodium tanshinone iia sulfonate injection" OR "tanshione")
	#3	TS=("clinical trial" OR "clinical stud*" OR "random*" OR "RCT")

	#4	#1 AND #2 AND #3
Cochrane Library	#1	MeSH descriptor: [Atherosclerosis] explode all trees
	#2	MeSH descriptor: [Cardiovascular Diseases] explode all trees
	#3	MeSH descriptor: [Acute Coronary Syndrome] explode all trees
	#4	MeSH descriptor: [Coronary Artery Disease] explode all trees
	#5	MeSH descriptor: [Coronary Disease] explode all trees
	#6	MeSH descriptor: [Cardiomyopathies] explode all trees
	#7	MeSH descriptor: [Stroke] explode all trees
	#8	MeSH descriptor: [ischemic attack, transient] explode all trees
	#9	MeSH descriptor: [Peripheral Arterial Disease] explode all trees
	#10	MeSH descriptor: [Myocardial Infarction] explode all trees
	#11	MeSH descriptor: [angina, stable] explode all trees
	#12	MeSH descriptor: [angina, unstable] explode all trees
	#13	MeSH descriptor: [Aortic Aneurysm] explode all trees
	#14	MeSH descriptor: [Cerebrovascular Disorders] explode all trees
	#15	MeSH descriptor: [Coronary Artery Bypass] explode all trees
	#16	MeSH descriptor: [Percutaneous Coronary Intervention] explode all trees
	#17	MeSH descriptor: [angioplasty, balloon, coronary] explode all trees
	#18	(atheroscleros*):ti,ab,kw OR (Atheroscleroses):ti,ab,kw OR (arteriosclerotic cardiovascular disease):ti,ab,kw OR (cardiovascular disease):ti,ab,kw OR (major adverse cardiac events):ti,ab,kw OR (acute coronary syndrome*):ti,ab,kw OR (coronary artery disease):ti,ab,kw OR (coronary heart disease):ti,ab,kw OR (revascularization):ti,ab,kw OR (ischemic cardiomyopathy):ti,ab,kw OR (stroke*):ti,ab,kw OR (Apoplexy):ti,ab,kw OR (transient ischemic attack*):ti,ab,kw OR (peripheral arterial disease*):ti,ab,kw OR (myocardial infarct*):ti,ab,kw OR (heart attack*):ti,ab,kw OR (stable angina*):ti,ab,kw OR (unstable angina*):ti,ab,kw OR (aortic aneurysm*):ti,ab,kw OR (cerebrovascular disease):ti,ab,kw OR (cerebrovascular disorders):ti,ab,kw OR (coronary artery bypass*):ti,ab,kw OR (aortocoronary bypass*):ti,ab,kw OR (percutaneous coronary

		intervention*):ti,ab,kw OR (percutaneous coronary revascularization*):ti,ab,kw OR (transluminal coronary balloon dilation):ti,ab,kw OR (coronary balloon angioplast*):ti,ab,kw OR (percutaneous transluminal coronary angioplast*):ti,ab,kw
	#19	(tanshinone iia):ti,ab,kw OR (tan iia):ti,ab,kw OR (TNA):ti,ab,kw OR (TSIIA):ti,ab,kw OR (Tansh):ti,ab,kw OR (sodium tanshinone iia silate):ti,ab,kw OR (STS):ti,ab,kw OR (sodium tanshinone iia sulfonate injection):ti,ab,kw OR (tanshione) :ti,ab,kw
	#20	(clinical trial):ti,ab,kw OR (clinical stud*):ti,ab,kw OR (random*):ti,ab,kw OR (RCT) :ti,ab,kw
	#21	#1 OR #2 OR #3 OR #4 OR #5 OR #6 OR #7 OR #8 OR #9 OR #10 OR #11 OR #12 OR #13 OR #14 OR #15 OR #16 OR #17 OR #18
	#22	#21 AND #19 AND #20
Ebsco	#1	SU atheroscleros* OR SU Atheroscleroses OR SU arteriosclerotic cardiovascular disease OR SU cardiovascular disease OR SU major adverse cardiac events OR SU acute coronary syndrome* OR SU coronary artery disease OR SU coronary heart disease OR SU revascularization OR SU ischemic cardiomyopathy OR SU stroke* OR SU Apoplexy OR SU transient ischemic attack* OR SU peripheral arterial disease* OR SU myocardial infarct* OR SU heart attack* OR SU stable angina* OR SU unstable angina* OR SU aortic aneurysm* OR SU cerebrovascular disease OR SU cerebrovascular disorders OR SU coronary artery bypass* OR SU aortocoronary bypass* OR SU percutaneous coronary intervention* OR SU percutaneous coronary revascularization* OR SU transluminal coronary balloon dilation OR SU coronary balloon angioplast* OR SU percutaneous transluminal coronary angioplast*
	#2	SU tanshinone iia OR SU tan iia OR SU TNA OR SU TSIIA OR SU Tansh OR SU sodium tanshinone iia silate OR SU STS OR SU sodium tanshinone iia sulfonate injection OR SU tanshione
	#3	#1 AND #2

1.1.2 Supplementary Table 2: The search strategy for Chinese databases.

**Database**	**Strategy**

CNKI	TKA=("动脉粥样硬化" + "动脉粥样硬化性心血管病" + "急性冠状动脉综合征" + "冠心病" + "慢性冠状动脉综合征" +"血运重建术" + "血管重建术" + "缺血性心肌病" + "中风" + "卒中" + "短暂性脑缺血发作" + "外周动脉粥样硬化疾病" + "外周动脉疾病" + "周围动脉疾病" + "主动脉瘤" + "心肌梗死" + "心绞痛" + "脑血管" + "冠状动脉") AND TKA=("丹参酮" + "丹参酮 IIA" + "丹参酮 IIA 磺酸钠") AND TKA=("试验" + "随机" + "对照" + "临床试验" + "随机对照试验")
VIP	(M=("动脉粥样硬化" OR "动脉粥样硬化性心血管病" OR "急性冠状动脉综合征" OR "冠心病" OR "慢性冠状动脉综合征" OR"血运重建术" OR "血管重建术" OR "缺血性心肌病" OR "中风" OR "卒中" OR "短暂性脑缺血发作" OR "外周动脉粥样硬化疾病" OR "外周动脉疾病" OR "周围动脉疾病" OR "主动脉瘤" OR "心肌梗死" OR "心绞痛" OR "脑血管" OR "冠状动脉") OR R=("动脉粥样硬化" OR "动脉粥样硬化性心血管病" OR "急性冠状动脉综合征" OR "冠心病" OR "慢性冠状动脉综合征" OR"血运重建术" OR "血管重建术" OR "缺血性心肌病" OR "中风" OR "卒中" OR "短暂性脑缺血发作" OR "外周动脉粥样硬化疾病" OR "外周动脉疾病" OR "周围动脉疾病" OR "主动脉瘤" OR "心肌梗死" OR "心绞痛" OR "脑血管" OR "冠状动脉")) AND (M=("丹参酮" OR "丹参酮 IIA" OR "丹参酮 IIA 磺酸钠") OR R=("丹参酮" OR "丹参酮 IIA" OR "丹参酮 IIA 磺酸钠")) AND (M=("试验" OR "随机" OR "对照" OR "临床试验" OR "随机对照试验") OR R=("试验" OR "随机" OR "对照" OR "临床试验" OR "随机对照试验"))
Wangfang Data	(题名或关键词:(动脉粥样硬化) or 题名或关键词:(动脉粥样硬化性心血管病) or 题名或关键词:(急性冠状动脉综合征) or 题名或关键词:(冠心病) or 题名或关键词:(慢性冠状动脉综合征) or 题名或关键词:(血运重建术) or 题名或关键词:(血管重建术) or 题名或关键词:(缺血性心肌病) or 题名或关键词:(中风) or 题名或关键词:(卒中) or 题名或关键词:(短暂性脑缺血发作) or 题名或关键词:(外周动脉粥样硬化疾病) or 题名或关键词:(外周动脉疾病) or 题名或关键词:(周围动脉疾病) or 题名或关键词:(主动脉瘤) or 题名或关键词:(心肌梗死) or 题名或关键词:(心绞痛) or 题名或关键词:(脑血管) or 题名或关键词:(冠状动脉)) and (题名或关键词:(丹参酮) or 题名或关键词:(丹参酮 IIA) or 题名或关键词:(丹参酮 IIA 磺酸钠)) and (题名或关键词:(试验) or 题名或关键词:(随机) or 题名或关键词:(对照) or 题名或关键词:(临床试验) or 题名或关键词:(随机对照试验))

1.1.3 Supplementary Table 3: Subgroup analyses of the influence of STS on IL-6 and TNF-α.

Variables	N	I^2 (%)	SMD (95%CI)	pvalue
**IL-6**				
Total	26	97	-1.50[-2.06, -0.95]	＜0.00001
Type of condition				
CAD	10	98	-0.70[-1.82, 0.42]	0.22
unstable angina	4	94	-3.85[-5.45, -2.25]	＜0.00001
AMI	4	93	-2.27[-3.25, -1.29]	＜0.00001
ACS	3	86	-1.61[-2.30, -0.91]	＜0.00001
ischemic stroke	3	99	-0.43[-2.91, 2.05]	0.73
AS combined with other disease	2	0	-0.62[-0.88, -0.36]	＜0.00001
Dosage of STS				
50 mg/d	2	0	-1.07[-1.30, -0.84]	＜0.00001
60 mg/d	10	96	-2.19[-3.12, -1.26]	＜0.00001
80 mg/d	11	97	-1.75[-2.53, -0.98]	＜0.00001
Unclear	3	99	1.84[-1.78, 5.46]	0.32
Duration				
1 w	7	95	-2.03[-2.23, -1.84]	＜0.00001
2 w	11	97	-0.79[-0.93,-0.64]	＜0.00001
3 w	3	86	-1.01[-1.26, -0.76]	＜0.00001
4 w	4	86	-3.11[-3.36, -2.86]	＜0.00001
12 w	1	—	-0.53[-0.86, -0.21]	0.001
**TNF-α**				
Total	25	98	-2.55[-3.24, -1.86]	＜0.00001
Type of condition				

CAD	12	98	-1.83[-2.93, -0.72]	0.001
unstable angina	1	—	-2.73[-3.35, -2.12]	<0.00001
AMI	5	98	-4.33[-6.34, -2.31]	<0.0001
ACS	5	97	-3.25[-4.80, -1.71]	<0.0001
ischemic stroke	1	—	-0.80[-1.07, -0.52]	<0.00001
AS combined with other disease	1	—	-0.68[-1.10, -0.25]	0.002
Dosage of STS				
40 mg/d	2	96	-6.97[-10.34, -3.60]	<0.0001
50 mg/d	3	97	-3.34[-5.14, -1.55]	0.0003
60 mg/d	10	95	-2.74[-3.46, -2.02]	<0.00001
80 mg/d	8	98	-2.19[-3.39, -0.99]	0.0004
Unclear	2	99	2.65[-3.91, 9.21]	0.43
Duration				
1 w	4	95	-3.25[-4.64, -1.86]	<0.00001
2 w	11	98	-2.05[-3.36, -0.74]	0.002
3 w	1	—	-0.68[-1.07, -0.29]	0.0007
4 w	8	98	-2.79[-3.93, -1.64]	<0.00001
7 w	1	—	-5.26[-6.21, -4.32]	<0.00001

ACS acute coronary syndrome, AMI acute myocardial infarction, AS atherosclerosis, CAD coronary artery disease, IL-6 interleukin-6, N number of SMD included, SMD standardized mean differences, STS sodium tanshinone IIA sulfonate, TNF-α tumor necrosis factor alpha, w weeks, X mg/d X mg daily, — not applicable

1.2 Supplementary Figures

1.2.1 Supplementary Figure 1

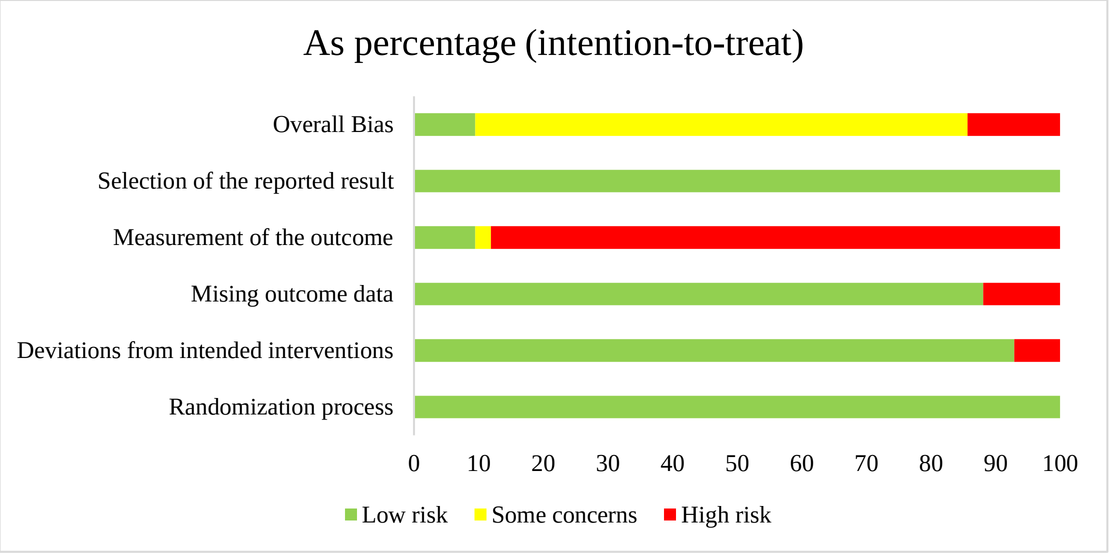

Supplementary Figure 1 Risk of bias summary.

1.2.2 Supplementary Figure 2

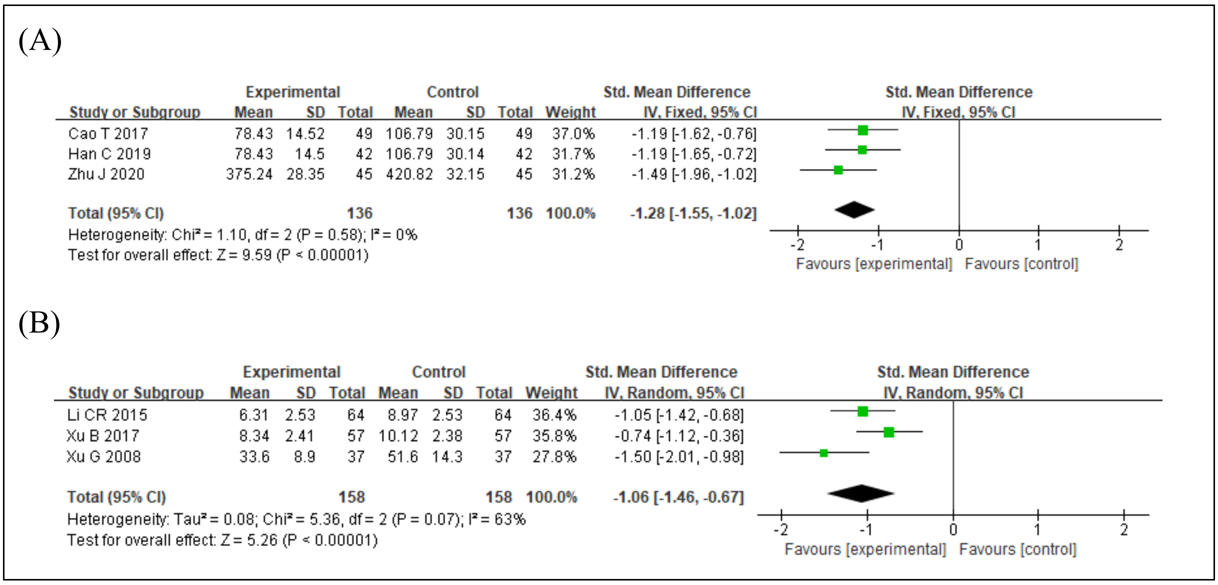

Supplementary Figure 2 Forest plots of the effect of STS on adhesion molecules: (A) ICAM-1 (B) p-selectin. ICAM-1 intercellular adhesion molecule-1, STS sodium tanshinone IIA sulfonate

1.2.3 Supplementary Figure 3

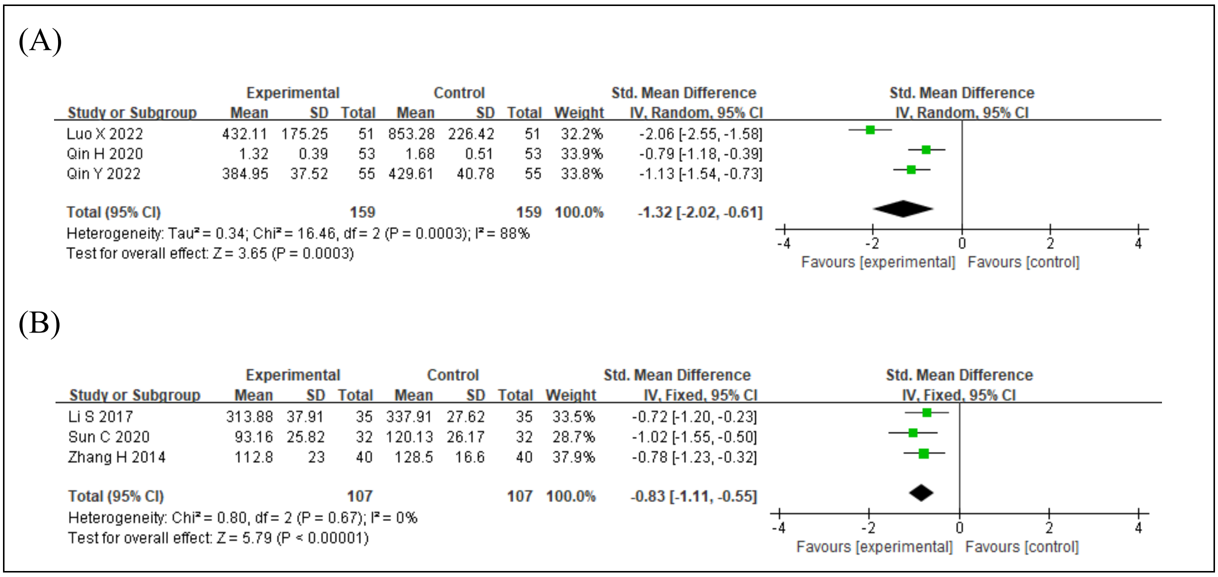

Supplementary Figure 3 Forest plots of the effect of STS on chemokines: (A) fractalkine (B) MCP-1. MCP-1 monocyte chemoattractant protein-1, STS sodium tanshinone IIA sulfonate
